# Theranostics: a multifaceted approach utilizing nano-biomaterials

**DOI:** 10.1186/s11671-024-03979-w

**Published:** 2024-02-26

**Authors:** Mohammad Yasir, Ratnakar Mishra, Alok Shiomurti Tripathi, Rahul K. Maurya, Ashutosh shahi, Magdi E. A. Zaki, Sami A. Al Hussain, Vijay H. Masand

**Affiliations:** 1 Amity Institute of Pharmacy, Lucknow, Amity University Uttar Pradesh, Sector125, Noida, 201313 India; 2 Era College of Pharmacy, Era University, Lucknow, 226003 India; 3 Department of Chemistry, College of Science, Imam Mohammad Ibn Saud Islamic University, Riyadh, 13318 Saudi Arabia; 4 Department of Chemistry, Vidya Bharati Mahavidyalaya, Amravati, Maharashtra India

**Keywords:** Biomaterials, Carriers of biomaterials, Carbon based materials, Imaging modalities, Theranostics

## Abstract

Biomaterials play a vital role in targeting therapeutics. Over the years, several biomaterials have gained wide attention in the treatment and diagnosis of diseases. Scientists are trying to make more personalized treatments for different diseases, as well as discovering novel single agents that can be used for prognosis, medication administration, and keeping track of how a treatment works. Theranostics based on nano-biomaterials have higher sensitivity and specificity for disease management than conventional techniques. This review provides a concise overview of various biomaterials, including carbon-based materials like fullerenes, graphene, carbon nanotubes (CNTs), and carbon nanofibers, and their involvement in theranostics of different diseases. In addition, the involvement of imaging techniques for theranostics applications was overviewed. Theranostics is an emerging strategy that has great potential for enhancing the accuracy and efficacy of medicinal interventions. Despite the presence of obstacles such as disease heterogeneity, toxicity, reproducibility, uniformity, upscaling production, and regulatory hurdles, the field of medical research and development has great promise due to its ability to provide patients with personalised care, facilitate early identification, and enable focused treatment.

## Introduction

Theranostic is a novel process that collectively explains the interrelationship of diagnostic imaging with treatment therapy. John Funkhouser interrelates two different but wide areas of the biomedical field, i.e., "therapeutics" and "diagnostics," and gave the new term "theranostic" in 1998. A.D. Theranostic is also called "Theragnostic." It was first used to test the efficacy of a new drug used as an anticoagulant [1].

In this process, a specific biological route is used to get a diagnostic image that shows the molecular target of an uncontrolled tumour cell and allows observation in real time. Then a therapeutic agent is delivered to the specific target receptor site of the tumour cell, which helps reduce off-target effects. The size range of colloidal nanoparticles (NPs) used in nanomedicine is 10–1000 nm in size. These nanomedicines are adsorbed, entrapped, conjugated, or encapsulated in macromolecular materials or polymers for theranostics at the cellular to molecular level [2].

To get desired diagnostic images, certain instruments, and techniques, such as optical fluorescence, bioluminescence, positron emission tomography (PET), single-photon emission computerized tomography (SPECT), Magnetic resonance imaging (MRI), ultrasound, Bioluminescence Resonance Energy Transfer (BRET), Förster resonance energy transfer (FRET), etc., are used. Theranostic technique is used in the treatment of many disorders such as thyrotoxicosis, thyroid cancer, and radiosynovectomy using radioisotopes such as lutetium, iodine-131, and yttrium-90, respectively [3]. In recent nanotechnology, carriers of NPs such as polymers, dendrimers, different metallic and nonmetallic NPs, carbon nanotubes, etc. are used to synthesize drugs in different modes such as controlled, immediate, or targeted delivery with minimum side effects [4]. Biomaterials are those materials that are formulated to interact with biological systems for the purposes of treatment and replacement. They can be natural or synthetic. Nowadays, biomaterials are used in theranostic fields as well due to their ability to give local and sustained effect. Among all the biomaterials used in theranostic therapy, the ligand-based theranostic model is the most preferred approach for the development of tumour cell-targeted biomaterials using the interaction of receptor and ligand [5, 6].

This review explains about theranostic nanomaterials the important theranostic properties of different NPs. Further it provides a concise introduction to the use of photothermal chemotherapy in the domain of cancer treatment, which incorporates the basic concepts of photothermal therapy (PTT). A summary of the utilization of carbon-based materials, including fullerenes, carbon nanofibers, graphene, and carbon nanotubes (CNTs), within the domain of theranostics is included. It also offers a comprehensive analysis of biomaterial carriers, including their present status, and their applications in theranostics. Furthermore, it is expected that theranostic systems will incorporate various imaging strategies for diagnostic purposes. These strategies include X-ray computed tomography (CT), nuclear positron emission tomography (PET), single photon emission computed tomography (SPECT), magnetic resonance imaging (MRI), ultrasound (US), optical fluorescent imaging, optical luminescent imaging, and photoacoustic tomography (PAT). By summarizing the state of the art in this field, the study gives valuable insights and suggestions for disease detection and therapy.

## Theranostic nanomaterials

Clinical diagnosis is a crucial step before treatment. Recently, scientists have been working to merge the processes of diagnosis and treatment. Following drug administration, clinicians can simultaneously image the treatment outcome and track drug localization. Theranostic nanomaterials are defined as fabricated nanoparticles used as dual-purpose materials for diagnostic and therapeutic purpose [7]. Theranostic NPs have been developed to transport various components and imaging agents in addition to their intended therapeutic activity, enabling simultaneous and synergistic diagnosis and treatments. Nanomaterials like polymer NPs, inorganic NPs and organic/inorganic hybrid nanomaterials can be used as a carrier as well as advanced theranostic agents, this use may lead to a reduction in harmful effects and an increase in good synergistic effects [8, 9]. With the aid of biomarkers and targeting ligands, advanced theranostic nanomedicine with multifunctional capabilities in nature has the capacity to detect disease and provide therapy to the affected cells. Small molecules, genetic materials, proteins, and peptides are some of the therapeutic agents used in theranostic nanomedicine.

Furthermore, Delivering the right doses of a therapeutic drug to the affected area of the body for a long time is known as targeted therapy in the treatment of disease [10]. Drug delivery systems (DDS) have been devised to manage the release of therapeutic agents to cure illnesses while also marking the damaged tissues or cells to provide active medications and imaging agents at the same time. To develop safer and more effective therapeutic value the nanomaterials are developed. Moreover, nanomaterials are prone to protein opsonization and aggregation as soon as they enter the bloodstream. The liver, spleen, and kidneys may use phagocytosis or filtration to remove the opsonized nanomaterials from the bloodstream. By lowering the retention duration, this rapid and extensive immune system clearance decreases the bioavailability [11]. To change the retention duration, the surface of the nanomaterials can be altered by adding polyethylene glycol (PEG), sugars, acetyl groups, or protein moieties. The contrast agent utilized is a key factor in the imaging methods used in theranostic [12]. Nanomaterials can be fitted for specific tasks by changing controllable physical as well as chemical characteristics like size, structure, ionization on the surface, solubility and pH to accurately regulate the pharmacokinetics of pharmaceuticals and imaging agents [13]. Carriers of nanomaterial used in theranostic are researched well before delivering in the body to improve its therapeutic effectiveness and potency during therapy [14].

## Important theranostic features of nanoparticles

Nanomaterials can be amorphous or crystalline; therefore, their electronic properties can be easily modified. The ideal characteristics of nanomaterials are equal strength, an active surface, and discrete energy levels. The suitability of NPs for drug administration and target increases with decreasing size. Due to their small size and mobility, NPs have a higher cellular absorption rate than microparticles, which enables them to target a variety of intracellular and cellular targets [15]. Because the targeted cells must internalize the NPs, the form of the particles is also important for biodistribution. Furthermore, they are better candidates for endosomal uptake, as immune system cells may recognize rod-shaped cationic NPs as rod-shaped bacteria [16].

The in vivo fate of NPs is also influenced by their hydrophobicity. Conventional non-modified NPs are susceptible to opsonization and are largely removed by the mononuclear phagocyte system [17]. The clearance and targeted administration of therapeutic NPs are both affected by their surface charge. There is a greater immunological response to positively charged NPs than to neutral or negatively charged ones. It has also been demonstrated that NPs having a surface potential between -10 and + 10 mV are resistant to phagocytosis and non-specific interactions [18]. The pH sensitivity of NPs is strongly correlated with their surface charge. NPs of this type can be programmed to identify and localize to specific subcellular locations. To get around this, the hydrophilic polymers or surfactants should be coated on them, or biodegradable copolymers with hydrophilic qualities, such as polyethylene oxide, PEG, poloxamer, polysorbate 80 (Tween 80), and poloxamine, should be used to create NPs [19, 20]. NPs contain special qualities including fluorescence characteristics (caused by the quantum effects of QDs) and the capacity to absorb and transport a variety of molecules, including medicines, proteins, and probes (due to their high surface to volume ratio as compared with the other macroscopic materials). The energy gap between the valence and conduction bands gets smaller as QD size grows, which gives QDs their peculiar fluorescence characteristics. This property allows them to absorb photons and emit a certain wavelength range between blue and red [21].

NPs are advantageous platforms for the target-specific and controlled delivery of micro- and macromolecules in disease therapy because of their ability to form stable interactions with ligands, their variability in size and shape, their high carrier capacity, and the ease with which they can bind both hydrophilic and hydrophobic substances [12]. Only NPs that can pass through the mucus coating the epithelium will be absorbed by the tissue. On the other hand, a nanocarrier's surface needs to have completely distinct characteristics for it to be able to efficiently absorb across epithelial membranes and permeate through mucus. The self-assembled NPs facilitate both processes, allowing for efficient oral administration of insulin. The NPs have an insulin and cell penetrating peptide nanocomplex at their centre, surrounded by a hydrophilic N-(2-hydroxypropyl) methacrylamide copolymer (HPMA) covering that can be dissociated [22].

NPs derived from natural polymers like chitosan, gelatine, albumin, and alginate, on the other hand, appear to be able to circumvent toxicity concerns while also significantly enhancing the efficacy of therapeutic substances. A matrix system, in which the matrix is consistently polymeric NPs, is considered. Polymeric NPs can be prepared in a wide variety of ways, allowing for precise regulation of the release characteristics of the therapeutic drugs they carry. This permits the transport of a more potent dose of the agents to the site of action. The specificity of therapeutic drugs in targeted tissue can be further increased by modifying and functionalizing the surface of polymeric NPs with a specific recognition ligand [23].

### Magnetic nanoparticles

Nowadays, MNPs are used for therapeutic purposes due to their biocompatibility, minimum toxicity, better imaging capability, characteristic features, and molecular-level response to the stimulus. These are prepared in many sizes and surface-coated with biocompatible coating agents for transporting abundant materials like drugs, antimicrobial agents, genes, and proteins. Furthermore, MNPs are also preferably used as a contrast agent in MRI for anticancer therapy [24].

MNPs used in theranostic are prepared with iron oxide nanoparticles (IONP) such as maghemite [Gamma-Fe_2_O_3_], magnetite [Fe_3_O_4_], and gadolinium [chelated forms of gadolinium complexes]. They have the unique property of decomposing in the body into oxygen and iron. When IONP with sizes ranging from 10 nm are synthesized, they exhibit a superparamagnetic property known as Superparamagnetic Iron Oxide Nanoparticles (SPION). SPION are primarily used to detect tumour growth in body tissues. They build up over and around the target site, increasing contrast and assisting in theranostic. Magnetite was approved by the FDA as the most preferred MNPs due to its superior characteristics. MNPs, which have organic or inorganic materials on their surfaces, are much better for diagnosis and therapy. Because they are stable and work well, they are used to improve the contrast and overall performance of MRI, which improves the resolution of tissue and cell imaging. They are also used to deliver drugs to the right place and to cause hyperthermia more effectively and accurately. In modern times, MNPs are combined with another NPs to do multimodal imaging. This makes the contrast stronger and helps find diseases early so they can be treated right away [25]. Elements such as nickel, iron, and cobalt are superparamagnetic or ferromagnetic in nature. Sometimes a combination of these elements is also used for theragnosis [26]. The technique for preparation and synthesis of MNPs, like Fe_3_O_4_ NPs, includes physical methods, the co-precipitation method, the wet chemical preparation method, and the metal-doped iron oxide method. MNPs physical properties (size) influence and change their magnetic properties. Fe_3_O_4_NPs of diameter less than 50 nm shows a superparamagnetic effect, whereas Fe_3_O_4_NPs of diameter less than 100 nm shows soft ferrimagnetism or ferromagnetism. MNPs can stick together and form clusters. To stop this from happening, the surface is changed with coatings or other methods, or the MNPs are combined with nanocarriers. Surface modification can affect the charge on a surface, its properties, sensitivities such as pH, temperature, and biocatalysts. PEGylated MNPs circulate in the blood pool for a long duration, can be used for long-term MRI processes, and help to enhance permeability and retention effects on irregular tumour vasculature. MRI is one of the most used imaging techniques in theranostics for diagnostic purposes. MNPs used in MRI help obtain high-resolution images of anatomical structures at the cellular and molecular level. Today, MRI is often used with other imaging methods to get more information and get around the limitations of a single imaging method. such as MR-PET imaging or MR-optical imaging. Some of the radioisotopes are also used with SPIONs for multi-modular imaging. When the isotope of copper [Cu^64^] is functionalized with SPIONs via the diphosphonate group and injected in footpads, after 3 h, the contrast increases in the lymph nodes and enhances the clarity of PET and MRI imaging. SPIONs are the most prioritized and preferred MRI contrast agents. They extend T2 and T2* effects. The innermost magnetic nuclei of SPIONs are magnetite (Fe_3_O_4_), hematite (α-Fe_2_O_3_) and maghemite (γ-Fe_2_O_3_). The coating materials preferably used are PEG, polyacrylic acid [PAA], zwitterions, proteins, or peptides. Other NPs, such as F^19^, manganese [Mn], and gadolinium [Gd], are also used in MRI contrast. Gd and Mn have a paramagnetic metallic characteristic, which can cause T1-weighted MRI contrast. Gd is chelated via acrylic chelating agents or macrocyclic chelating agents. and can be fused with other compounds such as silica NPs, liposomes, dendrimers, and hydrogels. Mn based contrast agents used in MRI helps in studying and learning brain and retina, neuronal movement, tumour [27]. While synthesizing IONP, aqueous solution is used, and co-precipitation is done with Fe(II) and Fe(III) precursors. For colloidal suspending ability during formulation, hydrophilic polymers are added and thus prevent aggregation. Drug moieties can be simply coupled to IONPs with a suitable and compatible coating and then used for the therapy of diseases [28]. As previously said, it has been shown that Fe_3_O_4_-PEG-(DA)-FA NPs may be used as a novel nanoplatform for dynamic enhanced dual-mode T1/T2-weighted MR imaging of arthritis and other diseases, including cancer [29].

**Fig. 1:**
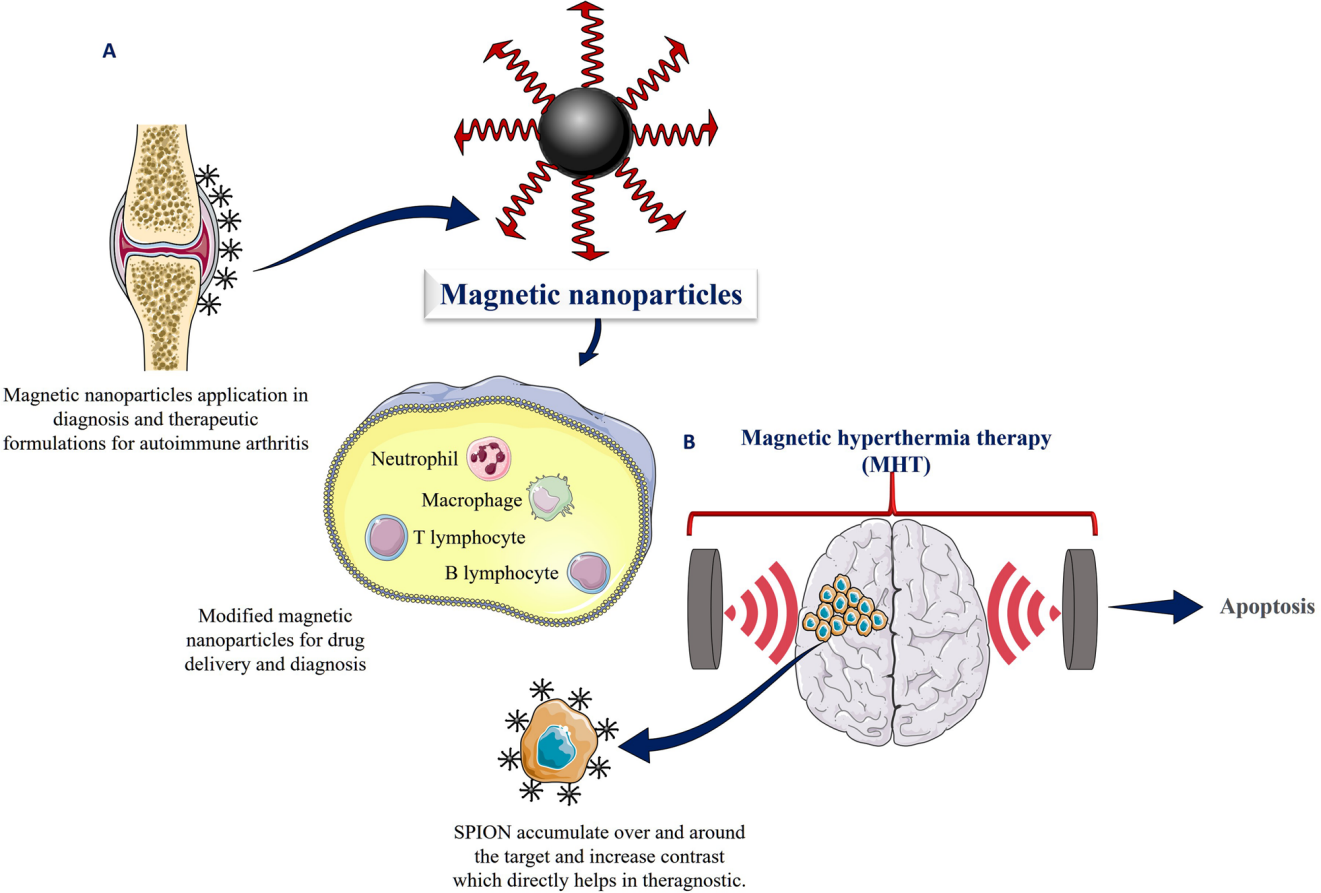
**a** Magnetic nanoparticles application in diagnosis and therapeutic formulations for autoimmune arthritis and surface modification of magnetic nanoparticles by various pharmaceuticals and biotechnological agents for the creation of multifunctional nanoparticles for theranostic applications **b** Magnetic nanoparticles are directed towards cancer cells utilizing the magnetic hyperthermia concept, resulting in restricted heating of cancer cells and death

Therapeutic components that are superficially attached to MNPs or encased in polymer, as well as MNPs composites, are used in magnetic systems for drug delivery. The importance of site-specific drug delivery includes the fact that it distributes medications without harming the body straight to the site of the ailment in a magnetic field outside the body while operating under a variety of conditions such as light, electric current, temperature, magnetic fields, and ultrasound Fig. 1A [25].

For the treatment of malignant tumours, Magnetic hyperthermia therapy (MHT) has been known to be effective since antiquity. Later, unorthodox approaches were utilized for tumour thermal ablation. The magnetic field is not absorbed by living tissues; the operating concept focuses on destroying the cancer cells by either apoptosis or necrosis. MHT works by using an alternating magnetic field to generate heat in a tumour that is made up of ferromagnetic NPs, resulting in the thermal ablation of the tumour tissue and causing the tumour to be destroyed. Magnetic hyperthermia has been used experimentally to treat cancerous tumours in the liver, prostate, breast, brain, and other organs Fig. 1B. Preclinical studies have also demonstrated that magnetic hyperthermia can be used to improve the efficacy of chemotherapy drugs by increasing the permeability of cell membranes and sensitizing cancer cells to chemotherapy [30]. Additionally, it has been suggested that magnetic hyperthermia can be used to enhance the efficacy of radiotherapy by producing a synergistic effect between the two treatments [31]. However, further research is needed to better understand the potential of magnetic hyperthermia therapy in combination with other treatments. Studies are needed to determine the optimum timing and dosages of magnetic hyperthermia in combination with chemotherapy and radiotherapy.

In contrast, there has been a growing focus on the use of immunotherapy in the field of cancer treatment in recent years, with generally positive outcomes being consistently documented. The utilization of MHT to stimulate the immune response against tumours holds promise in preventing cancer recurrence and metastasis, ultimately leading to a greater chance of survival. A study describing the use of a hybrid nanosystem (HNS) to suppress the growth and spread of tumours was reported. This HNS included the use of phase-transition nanodroplets that had immunomodulatory properties. These nanodroplets were employed to enhance the effectiveness of SPIONs in inducing MHT at temperatures below 44 °C. The fabrication process included the creation of magnetic-thermal-sensitive phase-transition nanodroplets by encapsulating the immune adjuvant resiquimod (R848) and the phase-transition agent perfluoropentane inside a shell made of PLGA. This HNS therapy has been shown to considerably reduce contralateral and lung metastasis via the combined effects of moderate MHT and immune activation [32]. A recent study documented the efficacy of using a combination of MHT enhanced cancer immunotherapy and transcatheter arterial embolisation (TAE) as a viable approach for the treatment of hepatocellular carcinoma (HCC). The uniform liquid metal microspheres (LM MSs) that possess strong eddy-thermal effects have the potential to serve as agents for both MHT and TAE, therefore offering a promising approach for the treatment of cancer. The efficacy of the eddy-thermal effect of LM MSs was established in achieving effective MHT. Additionally, LM MS-induced MHT was found to enhance the immune system, facilitate the infiltration of immune cells, and further activate robust immunological responses. These immune responses were seen to effectively limit the development of distant tumours when combined with immune checkpoint blockade therapy [33]. Another study which is related to glioblastoma reveals that MHT has the capacity to induce immunogenic cell death and enhance the susceptibility of glioblastoma multiforme cells to natural killer mediated cytotoxicity by upregulating specific stress ligands. This novel immunotherapeutic strategy holds promise for glioblastoma treatment and has the potential to synergistically enhance the therapeutic efficacy of existing NK cell-based therapies, thereby improving overall treatment outcomes [34].

MNPs were also involved with stem cell therapy. MNP-labelled stem cells were injected into organisms under a magnetic field to treat lung injury, atrial fibrillation, coronary artery embolism, vascular diseases, systemic osteoporosis, retinitis, high intraocular pressure, Parkinson's disease, myocardial infarction, and spinal cord injury, as well as regenerate articular cartilage [35]. These NPs can also be used to engineer tissues such as neural and cardiac tissues for applications in regenerative medicine [35]. Additionally, they can be used to guide transplanted cells to the site of injury, and thus improve the effectiveness of cell therapy.

In the field of chronic inflammatory conditions such as rheumatoid arthritis, MNPs demonstrated that when combined with therapeutics such as corticosteroids and dexamethasone acetate in Poly(lactic-co-glycolic) Acid (PLGA) microparticles, they can strengthen complex internalisation into synoviocytes and prolong drug action without cytotoxicity when an external magnet field was applied. This suggested that the magnetically enhanced delivery system could be an effective drug delivery method for both local and long-term rheumatoid arthritis treatment [36].

### Polymeric nanoparticles

PNPs are a type of nanomaterial designed for various medication delivery activities. PNPs can often be created by creating different useful units than solvent macromolecules; on the other hand, PNPs are sometimes formed by copolymer self-assembly. PNPs centres can be stacked with a variety of filling or display specialists in a common self-definition, where hydrolysis of the polymer network considers supported and controlled arrivals of these specialists. Also, the creation of drug particles in the polymer backbone allows for accurate drug stacking with extra control over the amount of drug released. To guarantee potency while limiting the immunogenic effect, the polymer channels are protected by stealth compounds like PEG [37, 38].

Dendrimers and polymeric micelles are globular nanostructures with amphiphilic copolymers and lipophilic centres that are used in polymeric controlled delivery and medication. These polymer frameworks focus on the possibility in contrast to conventional definitions, with the advantage of working with a vehicle to reach the target cell and the pharmacokinetics of cytotoxic atoms gaining comparable or improved viability in contrast to free medication, but with less or no harm [39].

**Fig. 2:**
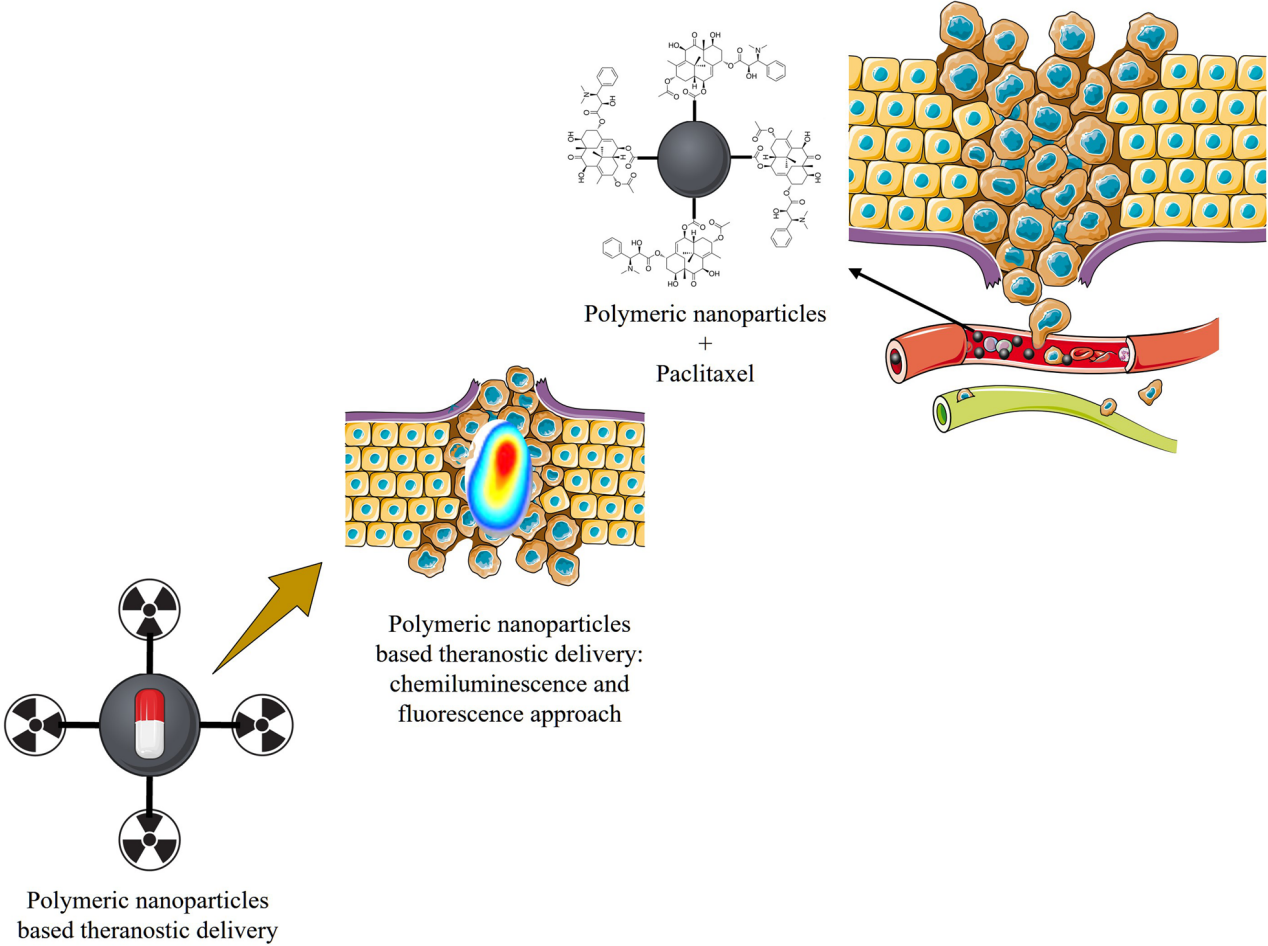
Demonstrating the potential of polymer conjugates with optically and electrically active materials as well as radiolabelled materials in biological imaging and medication delivery

Within the newest decade of eco-friendly polyurethane, it occurs and is also recognised as a dominant gatekeeper to aid healing, along with medicated visualization operative to facilitate and create many articulation possibilities. Several applicable synthetic resins of the small kind, such as polyurethane, use excellent ferromagnetic nano-dimensions as a basis, vortex ultrasonography, polyurethane nanoclusters of positional radioisotopes, and illuminating polyurethane nano-dimension design to achieve the goal based on nanomedicine. Each collective representation uses small categories and phrasing to approach scientific series in the coming time that could help clinicians predict treatment properties (for example, depending on the quantity of drugs collected at a harmful site) and record cancer development progress in patients who subsequently work on custom drugs Fig. 2 [38].

PNPs embedded dexamethasone prepared with PEG, used for the management of arthritis at lower dose. This micelle reduces bone erosion and swelling effectively, as it accumulates at joints due to long term availability [40]. Moreover, adverse effects of it are lower than the dexamethasone alone. Polycaprolactone (PCL) and dextran sulphate (DS) were prepared as an amphiphilic copolymer (DS-*b*-PCL), as one block hydrophobic and another hydrophilic block respectively. These NPs are used as carriers for anti-inflammatory drugs, which act on the synovial membrane by targeting macrophages receptors in the CIA mice [41]. These NPs are used for the management of some more disorders, A study reveals that paclitaxel (PTX) loaded PNPs effectively manage liver cancer in transplantable liver tumour-bearing mice, as it improves survival rate and inhibits tumour growth compared to paclitaxel plain tablet by improving bioavailability of it. Moreover, it was reported to be beneficial for the management of several other types of cancer [42]. There are several studies reported that antibiotics loaded PNPs shows improved antibacterial properties, as gentamycin loaded chitosan NPs were prepared for sol–gel systems ocular delivery by dispersing it into pH-sensitive Carbopol polymer solutions [43].

Even the most advanced cancers can be completely wiped out with the help of NIR multi-triggered drug release for successful PT chemotherapy. Overall, an engineered modular approach is used to make nanogels, which are common nanoplatforms that can be made to be responsive, flexible, and biodegradable for precise medical applications [44].

### Carbon based materials

**Fig. 3:**
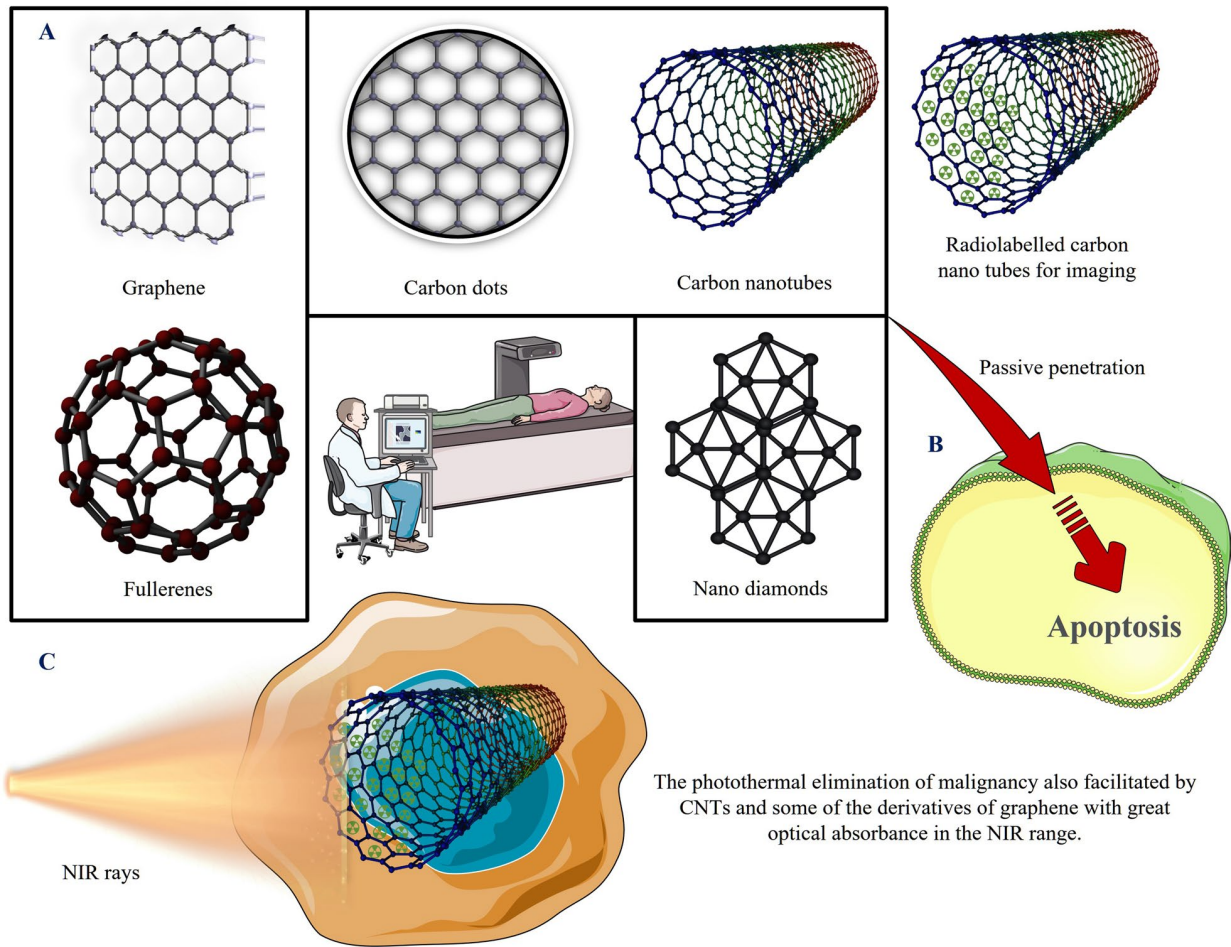
**a** Schematic representation of carbon-based nanomaterials. Examples of the use of carbon nanostructures can also serve in cell imaging due to fluorescence in the NIR region. **b** Carbon nanomaterials exerted their chemosensitizing effects by increasing apoptosis and suppressing proliferation. **c** Combining standard chemotherapeutics with carbon-based nanomaterials is an innovative way to treat cancer that makes it less likely that the patient will become resistant to the chemotherapeutics

Because the majority of carbon allotropes appear to be rarely cytotoxic and highly biocompatible with a wide range of substances, carbon is an ideal candidate for use as a nanocomposites in future nanomedicine and technological applications research [45]. Carbon-based materials such as fullerenes, carbon nanofibers, graphene, and carbon nanotubes (CNTs) have been continuously reported Fig. 3A. QDs, polymer-based NPs, and metallic nanocomposites were all intended to be replaced by carbon-based nanomaterials due to their better therapeutic activities. Nano-carbons (NCs) can be divided into sp^2^ and sp^3^ types of materials depending on the structure of the bond formed. Fullerene (0D), CNTs (1D), and graphene (2D) are examples of common sp^2^-carbon nanostructures. Carbon NPs, also known as carbon dots (C Dots), are amorphous nanoclusters of carbon with diameters less than 10 nm. Nanodiamonds (NDs) with nanoscale crystalline sizes are included in Sp^3^-carbon nanostructures. Numerous NCs, like graphene derivatives, C Dots, CNTs, and NDs, exhibit fascinating optical characteristics that make them useful as contrasting agents in imaging technologies. CNTs, as well as graphene, can be widely employed in a variety of biosensing technologies due to their superior electrical characteristics. Some of the CNTs and graphene, in which all the carbon atoms are revealed on their surfaces, can be used for effective drug packing and bioconjugation. The inside of CNTs and fullerenes has a hollow structure and therefore can be used to load different useful elements for theragnostic applications. The PT elimination of malignancy can also be facilitated by CNTs and some of the derivatives of graphene with great optical absorbance in the NIR range. Additionally, compared to numerous other inorganic NPs like QDs, which typically include heavy metals, NCs made of only carbon are relatively harmless in terms of their basic constitution [46].

#### Carbon nanotubes

CNTs are 1D carbon NPs that are divided into single-wall carbon nanotubes (SWCNTs) and multi-wall carbon nanotubes (MWCNTs), which are determined by the quantity of the graphene layer. It is possible to treat the sidewalls of both SWCNTs and MWCNTs as just a single sheet of graphene [27].

CNTs, which are rolled graphene layers shaped like needles, exhibit a broad range of mechanical and physicochemical characteristics. Because of their low solubility in water-based systems due to their hydrophobic characteristics, nanotubes are much more complicated to disperse uniformly than other nanomaterials. This makes it difficult to properly incorporate them with an active ingredient. To overcome this situation, they must go through some alterations to serve as carriers. The two techniques that can be used for its functionalization are exohedral and endohedral. The active ingredient is linked via covalent linkages or non-covalent linkages to perform exohedral derivatization. The second technique involves adding various polar chemicals to the space to fill the void. Because of their ease of penetrating cellular components, carbon nanotubes have opened the possibility of studying them as transporters for drug delivery systems in theragnostic. CNTs are used as a contrast material in MRI for diagnosis. The most popular area of study involved gadolinium chelates, which are added to CNTs. Because of their fluorescence in the NIR range, CNTs can also be used for cell imaging. The fact that CNTs have no effect on the life of diagnosed tissues is a major benefit. A particularly significant problem is the biodistribution and biodegradability of CNTs in the context of their prospective use in living beings. Additionally, the cytotoxicity of CNTs differs depending on their length.

Several studies have demonstrated that the longer the fibres, the higher the cytotoxic effect on tissues [47]. For targeted therapy, anti-cancer medications were joined to fused CNTs by 1,3-dipolar cycloaddition and amide linkages. CNT's distinctive physical characteristics make them useful for use in cutting-edge cancer treatments Fig. 3B. NIR areas exhibit considerable optical absorptivity for both MWNTs and SWNTs. CNTs produce a heating effect by absorbing light in the NIR region, which causes the thermal death of cancer cells Fig. 3C [48]. Due to their distinct electrochemical characteristics and stable chemical reactivity, these NPs make excellent choices as nanoprobes and bioimaging tools to identify viruses [14]. Mandal et al. investigated the effect of oxidized multi-walled carbon nanotubes (o-MWNTs) upon macrophages. It has been reported that subcutaneous injection of o-MWNTs will prevent tumour growth and metastasis by competing with malignant cells for recruiting macrophages from flowing monocytes, resulting in a decrease in macrophage density and lower vascular density in tumours. This discovery offers a brand-new method for employing CNTs in cancer immunotherapy [49]. A study reports on antibiotics loaded CNTs against multi drug resistant *Acinetobacter baumannii* and also improve wound healing compared to antibiotics alone [50].

#### Fullerenes

Fullerene has a particular geometry, shape, and surface properties, along with a spherical framework with highly nonpolar properties. Numerous chemical techniques are used to manufacture and functionalize a large number of fullerenes with unfilled carbon cages. This void inside can accommodate different atoms, including radionuclides that can be used in diagnostics. The C-60 and C-70 molecules are fullerenes that are most used. It's used in MRI as a new contrast medium, as well as pharmaceutical formulations and radioactive markers in PT treatment. Whether oxygen is present or not, functionalized fullerenes continue to glow under constant laser illumination. They exhibit very little photobleaching and have higher photostability. Following incubation, fullerenes can destroy tumour cells [47].

One method of using fullerenes in therapeutic and diagnostic medicine is to incorporate a Gadolinium (Gd) core into a fullerene. The Gd^3+^ ion is trapped within the pH of the body. the fullerene cage, which protects the metal ions' characteristics, prevents leaking, and nullifies in vivo disintegration. By the application of chemical alterations, the development of numerous varieties of "gadofullerenes" is possible. Li et al. encapsulated Gd^3+^ ions in fullerene and functionalized this with IL-13, which was then expressed on glioblastoma cells from humans. The molecule was chemically modified with NH_2_-groups, which may keep positive charges at physiological pH, to improve aqueous solubility and tumour uptake. Although this team of scientists hasn't yet reported any cytotoxicity tests, the initial tumour uptake and investigations on MRI are encouraging [51].

Chen et al. provide a thorough overview of the uses of synthesized fullerenes for tumour diagnostics. Fullerenes demonstrated promise in several tumour treatment modalities, including PT and photodynamic therapy, radiography, and chemotherapeutic treatment using imaging techniques. This also looks at the distribution, biochemistry, and cytotoxicity of fullerenes [52]. A report includes doxorubicin and cisplatin loaded C_60_ Fullerene NPs promotes cytotoxic effect by promoting antioxidant property and phagocytic action on lymphoblast [53]. Several reports reveal that phytochemicals loaded C_60_ Fullerene NPs attenuates diabetes and diabetes associated complications by modulating autophagy and apoptosis [54, 55]. It also has proven role against several virus including cytomegalovirus, Ebola virus, Herpes simplex virus, influenza virus and HIV[56–60].

#### Graphene

Graphene is a brand-new 2D graphitic carbon structure that has one atom of thickness. Graphene is extensively researched as a drug carrier and also for genes because of its extremely high total surface area, which is around four times greater than other nanomaterials studied for nanomedicines. Pure carbon is used to make graphene, and the atoms are assembled and structured, forming 2D honeycomb lattices, just as in graphite. Because graphene possesses stronger carbon–carbon bonds holding every carbon atom on the same surface together, it has significant conductivity both thermally and electrically and a small coefficient of thermal expansion. We can categorize the entire graphene family of nanomaterials (GFNs) based on different chemical changes, the quantity of layers, quality, and content. GFNs comprise graphene oxide (GO), reduced graphene oxide (R-GO), bilayer graphene, multilayer graphene, and single-layer graphene [47].

Graphene can be utilized for drug delivery vehicles. Because graphene and CNTs have comparable chemical structures, they can both be utilized as drug delivery vehicles. Loading of drug content on graphene surfaces with delocalized electrons is used to effectively load aromatic anticancer medicines like doxorubicin. The exceptionally high effectiveness of drug loading was made possible by the extremely huge total surface area of graphene on its surface [48]. GO has been used as a carrier system to carry a wide range of compounds, including antibodies, peptides, fluorescent dyes, anti-cancer medications, photosensitizers, siRNA, and SPECT agents [27]. Graphene becomes more sensitive when paired with other compounds and is used in pathogen identification and detection technology [14]. Its uses in nanobiotechnology have sparked a lot of curiosity. Graphene is used in biomedical fields such as anticancer therapies, bioimaging, and scaffolds for tissue engineering Shen [61].

Graphene NPs loaded with an anticancer drug (Doxorubicin) system improves anticancer properties of doxorubicin against oral squamous cell carcinoma [62]. A report reveals that graphene ameliorates inflammation, as reduces production of ROS and macrophages, it also altered the level of cytokines to promote the cardiac repair [63]. Graphene NPs also observed to reduce the development of bacterial resistance, as it also promotes the wound healing process [63, 64].

Graphene, a remarkable carbon material that exists in two dimensions, has generated considerable attention in the field of theranostics, which involves the integration of diagnostic and therapeutic capabilities. Graphene possesses distinctive physicochemical characteristics, including a large surface area, exceptional electrical conductivity, and compatibility with living organisms, which render it highly suitable for versatile theranostic platforms. Graphene-based materials have demonstrated significant promise as contrast agents in diagnostic imaging, encompassing modalities such as magnetic resonance imaging (MRI), computed tomography (CT), positron emission tomography (PET), and near-infrared (NIR) imaging. The remarkable imaging characteristics of graphene lead to improved resolution and sensitivity, effectively solving crucial issues in disease diagnosis and monitoring [65].

In addition to imaging, graphene is crucial in biosensing applications, providing fast and highly sensitive detection of biomolecules. Graphene-based biosensors possess exceptional specificity and selectivity, rendering them invaluable instruments for the prompt detection of diseases. Moreover, the incorporation of graphene with various transduction techniques improves the efficiency of biosensors, hence facilitating the advancement of sophisticated diagnostic instruments [66, 67].

Furthermore, graphene's adaptability may be shown in tailored drug delivery systems. Graphene-based systems enable targeted delivery tactics and controlled release, which have great potential for personalized medicine and better therapeutic outcomes. Hence, the diverse function of graphene in theranostics, highlighting its revolutionary influence on diagnostic applications and offering perspectives for future progressions [68–70].

#### Carbon dots

Carbon quantum dots (CQDs), also referred to as carbon quantum nanodots (C dots, are tiny carbon NPs with a diameter of fewer than 10 nm that had been mistakenly discovered by Xu et al. in 2004 while purifying SWCNTs [71]. They have been used as a drug carrier because of their minimal cytotoxicity, better biocompatibility, water solubility, and ease of customization. They can be prepared by various techniques, including chemically (with potent oxidizing acids), electrochemical carbonization, and laser ablation. Green luminous CQDs were made in less than a minute by microwave irradiation sucrose, which served as a source of carbon, and diethylene glycol (DEG). Photoluminescence (PL) is one of its most intriguing characteristics. As photosensitizers (PS), carbon nanodots can be utilized in PDT. Lanthanide hybridized quantum dots (Ln-CQDs) are also used as a bioimaging tool [47].

Sun and colleagues performed a study in which high-fluorescent CQDs without ZnS doping and with ZnS doping were synthesized and assessed for optical visualization systems in mice. Interestingly, CQDs exhibit competitive results in vivo compared to well-known CdSe/ZnS QDs, demonstrating that CQDs could be a novel category of optical imaging probes that do not comprise heavy metals and have potential for application in bioimaging [72].

CQDs reported for fast penetration through biological membranes, oral administration of CQDs loaded insulin reduces blood glucose level, as it acts on the mitochondria [73]. A study reports that CDs of thymol effectively manage rheumatoid arthritis, it reduces inflammation and improves the level of antioxidant enzymes blood parameters. Moreover, image analysis through ultrasonic image and X-ray analysis reported reduction of swelling, degeneration of joints and destruction of bone, which is evident that CDs have beneficial effects on rheumatoid arthritis [74].

#### Nanodiamonds

In nanomedicine, NDs with sp^3^-bonded structures have also received a lot of attention. Zhu et al. go through the cytotoxicity of NDs in both lab and real-life settings, as well as how they are used in medication delivery methods. The properties and characteristics of NDs and their clusters are quite distinct from those of CNTs and graphene, which makes them viable candidates for the delivery of drugs for fluorescent imaging-assisted delivery as well as reasonably safe in living organisms [75].

NDs has potential for drug delivery, which depends on surface chemistry, purity and other factors, and release of drug facilitated to get effective pharmacological effect. Huang et al. reported the cytotoxic effect of DOX loaded NDs on human colorectal cancer cells and mouse macrophages, reduces toxic effect due to sustain release of drug than free DOX [76]. Moreover, it shows beneficial effects on other types of cancer including liver and breast cancer [77]. *Escherichia coli* related urinary tract infection to the bladder tissue effectively kills by NDs, which depends on particle size as data of a study suggest that 6 nm NDs shows more reduction of *E. coli* bacteria on the bladder tissue than 25 nm size NDs [78].

### Quantum dots

Quantum dots (QDs) are biomaterials made of nanocrystalline semiconductors that can be used for both diagnosis and treatment. They act as nanoscaffolds in the process of theragnostic technique [24]. QDs have diameters ranging from 2 to 10 nm. Because of their special optical and electrical characteristics, QDs are used in many areas of biomedicine, such as therapy, diagnostics, and biosensing. They show several wavelengths and colours based on their physical structure. QDs remain at an excited energy level, which causes a loss of electrons. As a result, the core and the shell are covered with polymer. Furthermore, QDs are inorganic fluorophores with a low reaction to neighbouring compounds, resulting in increased photostability [14, 79].

During synthesis, the structure and size of QDs are affected by temperature, time, and the ligands used. Most of the QDs are prepared in a nonpolar organic solvent. If QDs must be solubilized in aqueous buffer solutions, amphiphilic ligands are used instead of hydrophobic surface ligands [45].

**Fig. 4:**
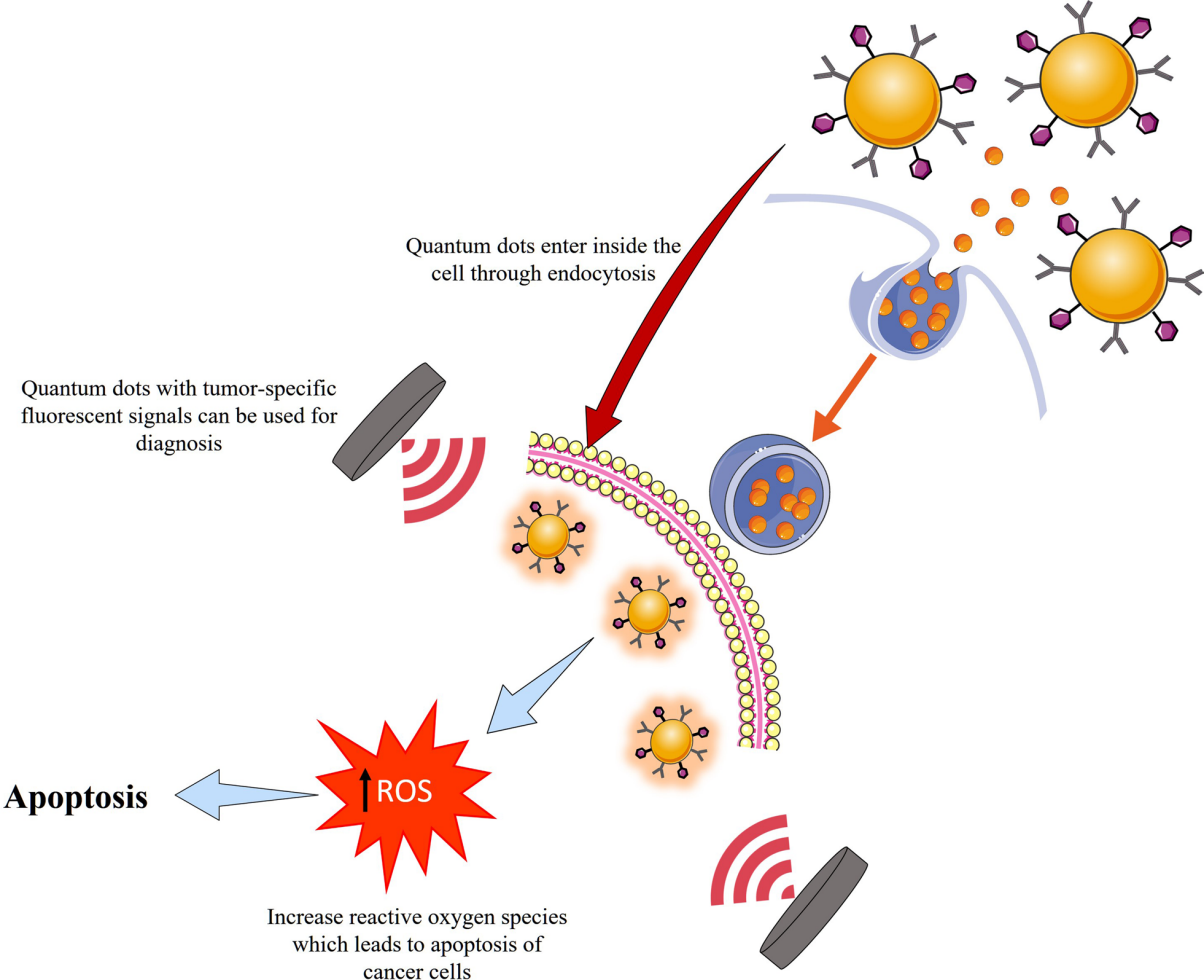
The low-density lipoprotein receptor/scavenger receptor and the G-protein-coupled receptor-associated pathway are the primary regulators of the quantum dots endocytic pathways. Nanoparticle absorption in mammalian cells for the purposes of cancer detection and therapy and medication administration is highly influenced by the nanoparticles' surface coating, size, and charge. QDs are effective energy givers; they transmit energy to oxygen molecules, resulting in the production of reactive oxygen species (ROS), which may then cause cell damage or death

QDs emit a narrower range of spectra and are more chemically stable. The optical properties of QDs can be managed by coordinating their composition with their dimensions. The largest generation of QDs were made from Pbs, CdTe, and CdSe, and by adjusting their size, they showed an increase in a variety of nanomaterials that span nearly the entire visible spectrum. Inorganic coatings like ZnS added to the surface of particles enhance the photoluminescent quantum efficiency of nanomaterials. The creation of QDs is similar to the IONP preparation process. To start making NPs, the right organometallic precursor compounds are heated to a high boiling point in an organic solvent. For the purpose of controlling particle development, surfactants such as trioctylphosphine oxide (TOPOx) and trioctylphosphine (TOP) are used. Similar to IONP synthesis, as synthesized QDs are alkylated and insoluble in water. The simplest method of granting solubility in water is to introduce thiolate-containing species to create disulfide connections with the QD core or ZnS shell. For such goals, a wide range of tiny thiolated compounds have been studied, including cysteine, glutathione, mercaptoacetic acid, mercaptopropionic acid, and mercaptosuccinic acid. A significant discovery is that, when QDs are modified with cysteine, they become hydrophilic with a hydrodynamic size smaller than 5.5 nm. Systemically administered modified QDs are now rapidly cleared by the kidneys rather than becoming lodged in organs such as the liver and spleen, as with other nanoformulations. A mercaptopropionic acid coating was applied to InAs, InP, and ZnSe QDs with an average size of 8 nm. Because of the inherent toxicity of QDs, QD-based medication delivery is understudied. Since poisonous cadmium [Cd] and lead [Pb] are widely employed in the manufacture of first-generation QDs, this issue is more noticeable. For the goal of improving QD manufacturing, Cd-free QDs like InAs/InP/ZnSe and InAs/ZnSe are becoming more popular. Nurunnabi et al., reported the preparation of QDs-Herceptin conjugates [80]. PEG-10,12-pentacosadienoic acid (PEG-PCDA) was employed to make the CdTe/CdSe QDs soluble in water. Then ultraviolet light was used to effectively stabilize the nanostructure and assist in restricting the possibility of Cd^2+^ leakage from core materials. When these NPs were evaluated on an MDA-MB-231 tumor model, they demonstrated effective tumour targeting rates and excellent therapeutic efficacy. Furthermore, with the help of optical as well as MR imaging techniques, optimal tumour targeting was seen Fig. 4. In one study performed by Yuan et al., he put MTX on the surface of QDs in order to cause quenching of photoluminescence (PL). The loading step was performed through simple physical adsorption that is reversible when introduced to molecules having greater affinities, like DNA. The PL was restored as a result of coating modification, which helps in medication delivery monitoring [81].

Additionally, QDs have great promise in therapeutic applications, where they serve as transporters or photosensitizers. The first paradigm's fundamental process is that, when stimulated using light, QDs transmit energy from the triplet state to nearby oxygen molecules, resulting in cellular destruction. When altered using lipofectamine or any other positively ionized polymer, QDs can also serve as gene carriers [28].

Certain QDs have the ability to convert light energy into thermal energy, enabling photothermal (PT) therapy (PTT). CdTe and CdSe QDs covered with SiO_2_ were utilized as therapeutic and diagnostic materials by Chu et al. The position of a QD in the malignant cells was identified by its fluorescence signals. Then the tumour was eliminated by the photo-thermal effects of laser light at 671 nm [82]. Ge et al. used graphene QDs in the treatment of breast tumours [83]. For the phototherapy of pancreatic tumours, Nigam et al. utilized graphene, which is conjugated with QDs [27]. Large amounts of quantum output, photostability, a high signal-to-noise ratio, and simultaneous stimulation of various wavelengths are advantages, whereas the formation of cytotoxic molecules and quick renal clearance are the drawbacks of QDs [26]. The alternative to QDs that is getting recognition is core–shell silica NPs (C dots) [84]. Recently, C dots were employed to identify tumour cells in order to investigate early bone metastases [6]. QD is also used to identify the H_1_N_1_ and H_3_N_2_ subtypes of the influenza A virus and to diagnose hepatitis B virus (HBV) [67, 68].

### Gold nano particles

Gold nanoparticles (AuNPs) are currently used in theragnostic as medication transporters, PT converters, visualizing probes, and radiosensitizers. AuNPs, which can be found in the shapes of nanospheres (2–100 nm), nanocages (40–50 nm), nanorods (10–100 nm), nanoshells (100 nm), and wires, are now obtained in huge quantities and with precise quality control (QC). AuNPs are a promising substrate in the biomedical field because of the chemical nature of gold. AuNPs have several distinguishing characteristics that set them apart from other substrates used in theragnostic [24, 85, 86].

Because of the surface plasmon properties of AuNPs, they have unique absorption spectra in the visible area. These traits are connected to the AuNP's morphology, size, and form. The availability of redox compounds, pH, and physical contact are examples of physicochemical factors that affect the free electron present on the surface of AuNPs. AuNP reactions can change coloration, which makes them ideal NPs for an optical signal-based diagnostic system. Another feature of these NPs is their adaptability to interface modification. The AuNPs may easily and effectively form covalent bonds with a variety of molecules of interest, including antibodies, antigens, and probes, due to chemistry of Au-thiol. Therefore, target-specific recognition with the normal eye is made easier by surface-modified AuNPs that are designed to attach to specific target entities. The interaction of AuNP with the target quickly causes a change in colour, and the optical responsiveness of AuNP is also visible [14].

AuNPs are studied in several imaging-related fields like surface-enhanced Raman spectroscopy (SERS), CT, and photoacoustic, and they possess a variety of distinct characteristics. Controlling the morphology of AuNP is crucial since it has a significant impact on its physical characteristics, which in turn significantly influence how well they function as visualization probes. For example, 10 nm spherical AuNPs exhibit surface plasmonic absorption at about 520 nm. The maximal absorbance wavelengths of 48.3 nm and 99.4 nm gold NPs are 533 nm and 575 nm, respectively. Modifying the NPs morphology to a rod shape can shift absorption to the near-infrared region (NIR) (650–900 nm), implying a function as agents in bioimaging or mediators of PT therapy. Functional compounds are loaded more frequently using the Au-thiol process. For example, PTX was altered at the 7th position of carbon (C-7 position) and then covalently joined to 4-mercaptophenol-modified AuNPs. The conjugates outperformed MTX alone in in-vivo as well as in-vitro. Protein-based medications were also mounted on AuNPs in a similar way. For example, when tumour necrosis factor (TNF) was combined with PEGylated AuNPs, the resultant conjugates showed greater treatment effectiveness and reduced toxicity than natural TNF. When AuNPs are collected around tumours, they can use PT therapy to kill nearby malignant cells by changing light energy into heat energy. Such a therapy model avoids normal cell damage by simply operating within the restricted illumination area. Spherically shaped AuNPs having a distinctive absorbance at 500–600 nm are not preferred for this purpose. On the other side, switching the design to a nanorod, nanoshell, or nanocage can move the absorption into the NIR range, and reports of AuNPs-based hyperthermia are increasing. AuNP are used as a potential material for creating functional substances for both diagnostic and treatment because of their special properties, which include significant surface plasmon absorption, durability, quality standards, and ease in customization. As a result, gold nanocomposites were employed in cancer therapy, immunization, tumour monitoring, and targeted medication administration [87].

**Fig. 5:**
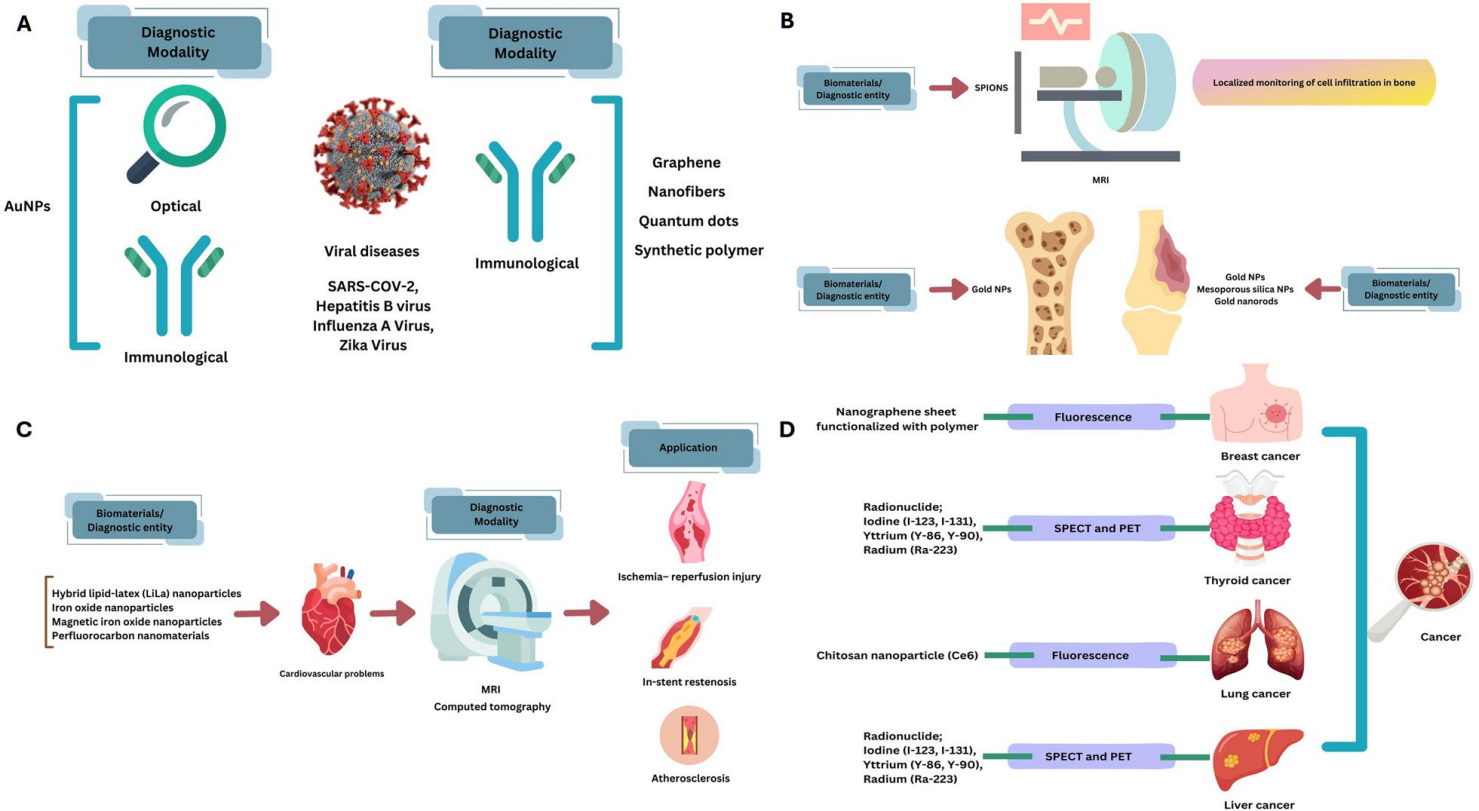
Schematic representation of multifaceted application of nano-biomaterials in the field of **a** Viral diseases **b** Orthopaedics **c** Cardiovascular problems **d** Cancer

Due to their superior X-ray absorption characteristics compared to iodinated CT imaging techniques, AuNPs were also employed as high-quality CT imaging techniques. Even though the gold centre is typically nonreactive, nontoxic, and has excellent biocompatibility, the production process for gold nanostructures, their physical and chemical characteristics (like size, morphological characteristics, and ionic strength), surface conjugates, dosing frequency, and pathways of administering can all cause significant toxic effects of AuNPs [85]. Chitosan was employed by Pokharkar et al. to create AuNPs by acting as a protective coating and reductant. The chitosan-AuNPs that were produced were extremely positively charged and reported to be particularly effective at loading insulin (53%). Such compounds were investigated in the diabetic model to reduce postprandial hyperglycaemia [88]. Further AuNPs coated with sialic acid which ceases attachment of the influenza virus to the host cells, reduces the development of drug resistance of the virus. Moreover, anti-viral activity of AuNPs depends on the size, larger the size of it than virus effectively inhibits attachment compared to smaller [73] Fig. 5A. There are several reports suggesting that AuNPs effectively inhibit the deposition of beta amyloid on the neuronal tissue, which could be effectively used for the management of Alzheimer's disease [89].

### Silica nanoparticles

Mesoporous SNPs used in theragnostic applications can improve the survival of tissue and cells [90]. By hydrolyzing and condensing tetraethyl orthosilicate (TEOS), SNPs can be created. Mercaptopropylmethoxysilane (MPS) and Aminopropyltrimethoxysilane (APS) are commonly used as co-precursors for functional group incorporation in molecules. These molecules co-coagulate with the matrix of TEOS to deliver thiol (SH) and amine (NH2) functional groups to the material surface. During particle development, it is simple to introduce any functional molecule within the nanosystem. This method has been used to link and merge organic dyes with the Gd-DTPA complex into the matrix of SNPs to produce optically as well as magnetically active substances. Silica lacks imaging properties, it is used as a safe substance in diagnostic procedures. They provide a great foundation that makes it simple to load a wide variety of treatment and imaging capabilities, which make them a suitable option for theragnostic uses [28].

They have exceptional biocompatibility, a naturally vast surface area, configurable size, and a large pore diameter. Mesoporous silica and nanosized organic and inorganic components are combined to quickly create multifaceted nonmedicinal platforms for simultaneous treatment and diagnostic uses. On the other hand, MSNs created using an acidic process can result in a large loading structure. It is possible to create organic or inorganic composites using neutral synthesis techniques. The forms of MSNs are being customized into various formations by altering the reaction circumstances. The morphological factors that influence in vivo activity are size and shape. It has been claimed that smaller MSNs are better at delivering medications than larger ones. These are also employed in the transport of genes. This complex was sturdy and kept the enclosed resources safe [91]. Dexamethasone loaded SNPs enhances drug delivery efficiency, inhibits inflammation in rheumatoid arthritis rat model, which recovers cartilage and shows anti-inflammatory effect due to sustained release of drug Fig. 5B [92]. SNPs also show beneficial effects against several other disease conditions including cancer, resveratrol loaded SNPs treating drug resistance cancer like prostate cancer, as it reduces the release of drug up to 40% than free resveratrol [93].

Despite the fact that SNPs could not naturally possess bioimaging or therapeutic properties, their high porosity makes them suitable for these applications. The morphology of SNPs is crucial for these applications. Numerous medical benefits can be attained by using SNPs. The first benefit is that nanomaterials may have their size, shape, and surface qualities precisely controlled by adjusting the chemistry [94, 95]. Silica malleable chemistry is crucial for achieving optimum biocompatibility and biodistribution [96]. There is growing interest in the possible medical benefits of mesoporous silica nanoparticles (MSNs). Drug delivery systems (DDSs) based on MSNs have several benefits over conventional drug nanocarriers due to their specialised mesoporous structure and large surface area. Due to its degradation to harmless silicic acid in vivo, spherical SNPs may be well tolerated by the body because this compound is already present in the body in a variety of tissues and may be eliminated via the urinary tract [61]. The potential for harm grows with increasing dose. Silicosis, which is brought on by exposure to NPs of crystal silica, is a serious health risk [97]. Due to its high biocompatibility, however, amorphous silica has been utilised as a food ingredient for decades. In most cases, SNPs store drugs within their mesopores and then diffuse those drugs out into the surrounding environment [98]. Because of this, stability is extremely necessary to ensure that the SNPs are able to be kept in the body and continue to release the medicine. This stability also ensures that there will be no leakage of the drug from the capped porous SNPs, which will release the medication in response to external stimuli. Furthermore, the vast surface area and porous architectures of SNPs are what cause their beneficial effects in medicinal applications. Because of the enormous surface area, there are plenty of conjugation sites or physical binding sites available for medicinal compounds. Additionally, the pores in the SNPs have the ability to physically store and protect payloads. SNPs can be produced in acidic, basic, or neutral environments [48, 99]. The fundamental synthesis is a base-catalyzed sol–gel process that includes two sub-steps, i.e., condensation and hydrolysis [100].

### Radioisotopes

Radioisotopes used in diagnostic and therapeutic procedures should generate beta radiation, which has a low permeability but a high capacity for cell damage, as well as gamma radiation. Additionally, visuals that can be utilized for diagnosis should be created using radioisotopes that have proper half-lives. The choice of suitable radioactive isotopes will be the most crucial step in the development of radionuclides during the therapy process. Radiation emission features, physical half-life (t_1/2_), effectiveness, decay, price, and accessibility of radioactive isotopes must all be addressed while developing radiopharmaceuticals [1].

Some of the radiopharmaceuticals and their isotopes like Iodine (I-123, I-131), Terbium (Tb-149, Tb-152, Tb-155, Tb-161), Copper (Cu-61, Cu-64, Cu-67), Arsenic (As-72, As-76), Yttrium (Y-86, Y-90), and Scandium (Sc-43, Sc-44, Sc-47), are utilized in theragnostic. The treatment makes use of charged particles such as (α-, β-, and Auger electron emitters, which solely focus on the target tissue and protect healthy tissue from needless radiation. The appropriate radioactive material and chelator can be employed to achieve this. Cancer cells are destroyed by the energy generated by radioactivity [101].

Beta and gamma emitters, Lu-177 and I-131, can be utilized for both therapy and imaging. Other isotopes of the same elements can also be used for theragnostic purposes. Examples include I-123 (a gamma emitter) and I-131 (a beta and gamma emitter). Terbium isotopes (Tb) such as Tb-152 (beta plus emitter), Tb-155 (gamma emitter), Tb-149 (alpha emitter), and Tb-161 (beta minus particle) are more good examples [24]. To facilitate tumour imaging with dual-mode SPECT/CT, folic acid (FA)-modified, (99 m)Tc-labelled, dendrimer-entrapped gold nanoparticles (Au DENPs) were developed [102].

The development of effective therapeutic radiopharmaceuticals requires the selection of the appropriate radionuclide and pharmacological component that can be merged in the specific part of the body to deliver the required amount of medication [1]. Radiopharmaceuticals used for different therapies depend on two major factors, 1st factor: physical parameters like effective half-life, purity, method of production, energy radiation and emission which promotes linear energy transfer (LET) and medical internal radiation dosimetry (MIRD); 2nd factor: biochemical factor toxicity, stability, retention in tumour and tissue targeting. Drug embedded radioactive NPs promote the disease tissue targeting by enhanced retention and permeability; permeation occurs due to leaking vasculature of tumour, promoting gaps to the endothelial cells of angiogenesis. Retention occurs due to alteration in lymphatic drainage, which retains the drug to the tumour tissue, leading to targeting overexpressed receptors of tumour cells that contribute in effective management of cancer [103].

Photothermal Therapy

Photothermal chemotherapy is a novel and promising strategy within the field of cancer treatment that integrates the fundamental principles of photothermal therapy (PTT) with conventional chemotherapy. The primary objective of this synergistic method is to optimize the therapeutic impact on cancer cells while concurrently limiting harm to healthy tissues, hence enhancing treatment success and mitigating adverse effects [104–106]. PTT serves as the fundamental principle underlying photothermal chemotherapy. The process is dependent on the utilization of light-absorbing substances or NPs, such as gold nanorods or carbon nanotubes, nanocages, silver NPs, graphene quantum dots (GQDs) that possess the ability to transform absorbed light into thermal energy [100, 101]. The light-absorbing compounds are supplied in a targeted manner to cancer cells. Near-infrared (NIR) absorbents are employed to enhance the efficacy of heat generation [107]. NIR light spectrum typically ranges from 700 to 1000 nm, and the transparency of biological tissue within this range makes it highly suitable for applications such as optical imaging and phototherapy [108]. Recent research has focused on investigating techniques to improve the targeted accumulation of drugs within a specific lesion by integrating a drug delivery system (DDS) with a NIR absorbent material. The implementation of a drug delivery system (DDS) has been found to enhance the efficacy of heat generation in tumour tissue, while simultaneously decreasing photothermal harm to adjacent normal tissue [109]. This advancement holds the potential to facilitate dependable PTT with heightened efficiency and exceptional safety.

One distinguishing characteristic of photothermal chemotherapy is its integration with conventional treatment. Chemotherapeutic agents are employed in the management of cancer to selectively target and eradicate actively proliferating cancerous cells [110–112]. However, it is noteworthy that these medications frequently exert an impact on non-malignant cells as well, hence giving rise to undesirable effects sometimes referred to as side effects. Precise guidance of PTT can be achieved by the utilization of fluorescence imaging, which enables control over laser positioning and tumour delineation. Additionally, photoacoustic imaging can be employed to delineate tumour margins and analyze the effectiveness of PTT doses, hence enhancing therapeutic efficacy [113, 114]. In the context of PTT, the generation of localized hyperthermia serves multiple significant functions. Therefore, it makes it easier for therapeutic agents to get to the cancer cells by increasing the permeability of the blood vessels inside the tumour.

Hyperthermia has the potential to attenuate or sensitize neoplastic cells, rendering them more vulnerable to the cytotoxic effects of chemotherapy [115]. This method works together to help lower the amount of medicine that needs to be given, which lowers the risk of systemic side effects.

## Carriers of biomaterials

In terms of medication delivery, image analysis, and diagnosis, nanocarriers (NCs) have emerged as one of the most potent and effective techniques. Organic carriers (such as dendrimers, micelles, and liposomes) as well as inorganic carriers (such as metallic nanocrystals, porous or hollow nanostructures, and carbon nanostructured materials) are frequently used nanocarriers that can be filled with contrasting agents and therapeutic entities via physical encapsulation and cross-linking of chemicals. The nanosized carriers that physically enclose functional cargoes present internally do have pore structures to allow loading, although not all cargoes must be entrapped inside the carriers throughout their construction. Covalent bonds, ionic forces, and hydrogen bonds, among other intermolecular interactions, may be used in the loading procedure. The carrier's primary duties include delivering therapeutic and diagnostic containments to the target location as covertly and selectively as possible while minimizing undesirable side effects caused by premature medication release. The system must be biodegradable and small, with a large surface area and the ability to change the surface's properties. They're quickly becoming an intriguing and successful drug delivery method [27, 116].

Some benefits of nanocarriers include greater effectiveness, site-specific targeting inside solid tumours or cancers from leaky vasculature, increased safety of antitumor agents by the use of non-toxic or biocompatible polymeric materials that restrict discharge of therapeutic material within normal tissues, improved water solubility of hydrophobic drugs, and making them suitable for parenteral administration [117]. Thus, it is anticipated that the construction of prodrug nano-systems could potentially powerful therapeutic strategies [118].

### Liposomes

It is composed of one or more lipid bilayers that are concentric and have an aqueous section. Liposomes are well-known medication delivery systems for cancer treatment. These are about 100 nm in diameter and are spherical vesicles enclosed in a lipid bilayer membrane with artificially synthesized or naturally occurring phospholipids. Someone with single bilayers is often created by hydrating thin lipid layers and then extruding or sonicating them. Drugs can be enclosed in liposomes using a variety of techniques because, as a result of the synthesis processes, the centre of the liposomes is occupied by an aqueous phase. In light of the fact that several liposome-based medicines, like Doxil®, have already received approval for the therapy of specific tumours, packing these carriers with therapeutic and diagnostic probes is a viable way to create a platform for next-generation nano-diagnostics [119–121].

For MRI purposes, a wide range of contrast chemicals like SPIOs, gadolinium, and manganese can be incorporated into liposomes, in particular manganese, gadolinium, and SPIOs. Liposomes can create nano-theranostics using radionuclide imaging by directly encapsulating radioactive agents. In order to create radiolabelled liposomes for tumour chemo-radionuclide therapy, Soundararajan et al. directly loaded rhenium-186 and doxorubicin into the interior of the liposome to create radiolabelled liposomes for tumour chemo-radionuclide therapy [37, 122].

Liposomes are among the most therapeutically applicable nanocarriers because of their good biocompatibility, consistent and large loading efficiency, and clinical applicability. There are also numerous ways to modify liposomes for more effective medication delivery and more focused targeting. When carriers are extensively steady, medication release and its availability at the target location will be less than ideal. Premature payload release brought on by the instability of liposomes is deleterious [123, 124].

To create modified liposomes known as porphysomes, porphyrin molecules were directly attached to lipids. Contrary to conventional encapsulation, porphyrin molecules were integrated into the nanoscale carrier. Porphysomes naturally have theranostic properties. Additionally, these new types of porphyrin-containing liposomes can reach various levels of theranostic efficacy by being packed with some other medications [113].

### Polymeric nanoparticles

A family of nanocarriers known as PNPs has been developed for several applications, including drug delivery. NPs can be created through the self-assembly of a co-polymer or by conjugating numerous functional subunits to soluble macromolecules. Various medicinal or imaging contrasts can be placed in the core of polymeric NPs in self-assembled formulations, in which the hydrolysis of the polymeric matrix enables their prolonged and controlled release. Additionally, accurate drug loading and further control of drug release profiles are made possible by conjugating drug molecules into the polymer backbone [125, 126].

Polymeric cores are protected by stealth substances like PEG to maintain stability while reducing immunogenicity. Additional targeting moiety can be added to the surface. In order to create polymeric NPs with greater biocompatibility, scientific research has been done on polymers made from natural resources, including cyclodextrin and chitosan [120, 121]. Polymeric micelles are produced from self-assembling amphipathic molecules in water. Micelles can directly conjugate or interact with other molecules to contain a variety of diagnostic and therapeutic cargoes. Hydrophobic chemicals can be trapped inside the centre of amphiphilic block copolymers, forming micelles that can solubilize a cargo [127].

PNPs can now be created using a broad variety of synthetic polymers with exceptional biocompatibility. The development of theranostic platforms has increasingly focused on polymer-based NPs. It has been demonstrated that polymeric NPs are efficient carriers of MRI contrast agents, including Gd-based chemicals and SPIOs [37]. Radionuclide imaging has been investigated using formulations based on polymers. Researchers have deeply investigated a huge variety of copolymers, including HPMA, to develop reliable nano-sized delivery models, for instance by conjugating radioactive compounds like C-11, F-18, Cu-64, Br-76, Y-90, Tc-99 m, and In-111 [128].

### Dendrimers

Macromolecular dendrimers have a branching structure all around them. They can be used theranostically for multiple purposes, including imaging and contrast, biotherapeutics, radiotherapeutics, chemotherapeutics, phototherapeutics, etc. [24]. In contrast to certain other nanocarriers, these are polymeric, hyper-branched, and have clearly defined chemical properties. Monomers can be combined to create spherical nanostructures with a central core that can contain medicinal drugs as well as hooks on their exterior surface for cell identification tags, enzymes, fluorescent dyes, and other compounds. Dendrimers are attractive nanocarriers for theranostic applications such as gene transfer, bioimaging, and tumour therapy due to their well-defined architectures and excellent cargo carrying ability and capacity. However, in order to advance biomedical studies, biocompatibility and long-term carrier clearance must be addressed [129, 130].

### Hollow and porous nanostructure

There are so many inorganic nanosized carriers that feature porous or hollow architectures. AuNPs with a porous centre, calcium phosphate NPs, mesoporous SNPs, CNTs, etc. are some of the examples. Graphene and CNTs have both developed into mesoporous materials. In order to be covalently connected with theranostic payloads, the surface of CNTs could be modified with various organic functional groups, like -COOH or -OH. Medicines can also be absorbed through molecular interactions such as stacking. These modifications can improve the solubility and biocompatibility of hollow materials for biomedical applications [27, 131].

## Imaging strategies in theranostic system

There are many imaging modalities that are used for diagnostic purposes, including CT (X-ray, computed tomography), nuclear PET, single photon emission computed tomography (SPECT), MRI, Ultrasound (US), optical fluorescent imaging and optical luminescent imaging, and photoacoustic tomography (PAT). These imaging techniques help in characterizing and monitoring biological activities at the tissue, cellular, and subcellular scales in living things. In some circumstances, imaging can monitor and explain disease progression in a therapeutically meaningful way by employing molecular contrasting chemicals or probes. For the purpose of designing automated algorithms and standards for diagnosing non-invasive medical image analysis, quantitative measurements of contrasting agents are needed. The right combinations of several imaging technologies help maximize efficiency and support the development of new diagnostic approaches. It would be ideal to create a single contrasting agent that can work with several imaging techniques Fig. 5C [3, 27].

The imaging techniques result in the confirmation of the presence of surface-specific receptors on target-specific sites and accurately calculate the incorporation of another drug carrier biomaterial. Monitoring of drug delivery carriers is also crucial since it can demonstrate that the cytotoxic element is successfully delivered and retained in the tumour. These diagnostic data are also utilized to associate delivery and treatment results, as well as to confirm their usage as imaging response indicators. The main drawbacks of optical imaging in deep body tissue include strong tissue absorbance and scattering; however, this problem can be somewhat resolved in preclinical imaging utilizing NIR dyes (Table 1) [132]. Indocyanine green (ICG) is a frequently used NIR dye that has found widespread application in NIR bioimaging, as well as PT and photodynamic treatment. Indocyanine green-human serum albumin NPs labelled with technetium-99 m (99mTc) demonstrated remarkable efficacy in facilitating preoperative planning through the utilisation of SPECT imaging in tumour metastasis. Additionally, these NPs enabled intraoperative real-time monitoring through NIR fluorescence imaging, as well as facilitated photothermal treatment of tumour metastasis. The use of photothermal therapy resulted in an augmented suppression of lung metastasis and a notable extension of the life period in mice. The produced NPs had a high level of biocompatibility and shown effective theranostic characteristics in the inhibition of cancer spread Fig. 5D [133]. NIR irradiation may lead to false positives in diagnostics and diminished treatment efficacy due to ICG accumulation outside of tumours in the liver, spleen, and kidneys. A hybrid nanomicelle was developed by merging hypoxia-sensitive iridium(III) and ICG for accurate tumour localization and photothermal treatment in succession. The experimental findings showed promise for therapeutic applications due to selective localization of nanomicelles to the tumour first [134]. Another study which is reported is related to the treatment of glioblastoma utilizing ICG. A unique theranostic nanoagent (NA) composed of Gd_2_(WO_4_)_3_:Nd^3+^ NPs loaded with ploy-L-arginine, ICG, and lactoferrin is designed. This nanoagent works on reactive nitrogen species (ROS) and reactive nitrogen species (RNS) pathways to make dual-modal imaging and effective treatment for glioblastoma easier to combine. The loading not only boosts the capacity of NA to create nitric oxide, block cell migration, and promote ROS/RNS for tumour eradication, but also results in the production of peroxynitrite with higher toxicity and a longer duration [135].

**Table 1:** Involvement of biomaterials in treatment of various diseases

Disease/Disorders	Biomaterials/Diagnostic entity	Diagnostic modality	Clinical application	References
Orthopaedics	SPIONS	MRI	Osteoporosis	[136]
	Carbon nanotubes (99mTc labelled carbon nanotubes)	Photoacoustic imaging	Active bone metabolism	[137]
	Quantum dots	NIR	Targeted cell imaging, BONE tumours	[138]
	Gold NPs	CT, Photoacoustic imaging	Damaged bone, Osteosarcoma	[139, 140]
	Mesoporous silica NPs (Mesoporous silica NPs loaded with BH_12_N_3_O_3_, Mesoporous silica NPs/ gold nanorods)	CT, Photoacoustic imaging	Osteosarcoma, Bone metastasis	[141, 142]
	Hydroxyapatite (Calcium deficient hydroxyapatite scaffold labelled with NIR probe, Nano-hydroxyapatite rods/ Molybdenum oxide, Iron-doped hydroxyapatite alginate-gelatine scaffold/ Bone morphogenic protein 2 (BMP2))	NIR, fluorescence, MRI	Monitor bone healing, bone infection, Localized monitoring of cell infiltration in bone	[143–145]
	Upconversion nanoparticles (UCNPs) (18F labelled NaGdF4:Yb, Er UCNPs), (Yb(III)/Ho(III) doped UCNPs) (NaYF4:Yb3 + ,Er3 + UCNPs)( NaYbF4:Gd3 + /Er3 + UCNPs)	MRI/PET, Luminescence, Fluorescence, Gemstone spectral CT	Bone, bone regeneration, cell localization, damaged bone	[28, 146–148]
	Polymer-based systems (99mTc labelled polymers, SPION coated with chitosan-PEG copolymer)	PET, MRI	Bone metastasis	[149, 150]
	Lipid-based systems (64Cu-porphysomes)	PET, fluorescence	Bone metastasis	[151]
Viral diseases	Carbon nanotube	Rapid Diagnostic Test,	Detect Dengue Virus, SARS COV-2,	[152, 153]
		Immunological	Influenza-A-Virus (H1N1)	[154]
	AuNPs	Optical	SARS-COV-2, Hepatitis B virus	[155, 156]
		Immunological	SARS COV-2, Influenza A Virus, Zika Virus	[157–159]
	Graphene	Immunological	Japanese Encephalitis Virus, Avian Influenza virus, HIV-1, Influenza A Virus (H5N1), Zika Virus	[160–164]
	Quantum dots	ELISA	Influenza-A Virus, SARS COV-2	[165, 166]
		Immunological	Hepatitis-E-virus-3	[167, 168]
	Nanofibers	Immunological	SARS COV-2	[169]
	Synthetic polymer	Immunological	Influenza A Virus (H1N1)	[170]
Cardiovascular problems	QDots (Qdot® 705 ITK™ amino PEG quantum dots) (Qdots 800 near infrared (NIR)(fluorescence)	Fluorescence/NIR	atherosclerosis	[171]
	Iron oxide NPs (AF750)	NIR, fluorescence	atherosclerosis	[172, 173]
	Iron oxide nanoparticles	MRI	Thrombotic diseases, injured cardiomyocytes	[174, 175]
	AuNPs (Cy 5 -Fluorescence)	Fluorescence	Myocardial infarction	[176]
	Copolyoxalate polymer nanoparticles (Rubrene)	Fluorescence	Ischemia– reperfusion injury	[177]
	Perfluorocarbon nanomaterials	MRI	In-stent restenosis	[178]
	Magnetic iron oxide nanoparticles	MRI	Vascular regeneration in the stent position	[179]
	Gold Nanorods	Computed tomography	Ablation of inflammatory macrophages	[180]
	Single walled carbon nanotubes	NIR/fluorescence imaging	Macrophagic apoptosis	[181]
	Hybrid lipid-latex (LiLa) nanoparticles (Rosiglitazone)	MRI	Targeting proliferation macrophages in atherosclerosis	[182]
Cancer	Polymer; Polyether’s (e.g., PEG-based) (PT agent (IR-780), folic acid)	–	Diagnostics therapy in Ovarian, colon, breast, lung	[183–187]
	AuNPs (Silica core coated with Au shell)	computed tomography, photoacoustic imaging	neck and head tumour	[188]
	Chitosan nanoparticle (Ce6)	Fluorescence	A549 lung cancer	[189]
	Polymeric nanoparticle with ESION (PDT (Ce6))	MR contrast	CT26 colorectal cancer	[190]
	Iron oxide nanoparticles (Azademethylcolchicine)	MR contrast, fluorescence	MMTV-PyMT lung cancer	[191]
	Nanographene sheet functionalized with polymer	Fluorescence	Breast cancer	[192]
	Mesoporous silicon nanospheres	Ultrasound/Fluorescence	Pancreatic cancer	[193]
	Maghemite NPs coated with rhodium (II) citrate	MRI	Drug carrier in breast Cancer	[194]
	Gold nanoclusters as radio sensitizing agents	computer tomography	Prostate cancer	[195]
	Zn-doped TiO2 nanoparticles	MRI	Breast cancer	[196]
	SPIONs coupled with PSMA	MRI	Prostate cancer	[197]
	Radionuclide; Iodine (I-123, I-131), Yttrium (Y-86, Y-90), Radium (Ra-223)	SPECT and PET	Thyroid cancer, liver cancer, metastatic prostate cancer in bones,	[24]

### Optical imaging

Optical imaging is a popular visualization technique in the preliminary investigation of NPs used in theranostic. These methods typically track probe-emitted photons after absorbing light waves. This method is less expensive than some other imaging techniques and does not expose users to radiation-related risks, but it still has certain serious disadvantages. Contrasting agents are frequently stimulated by both visible and UV light, which cannot invade deeply into tissues. The radiated fluorescence signals are also susceptible to noise because of scattering photons from nearby tissues or proteins that absorb radiation, such as heme in RBC. In this situation, NIR (range 650–1000 nm) fluorophores look promising by lowering autofluorescence and photon scattering to enable greater tissue penetration, which makes optical imaging more appropriate for in vivo testing. However, near-infrared imaging is restricted to tissues with very modest depths, making it ideal for imaging rodents. Even if it has been demonstrated that NIR-active NPs are capable of preclinical cancer detection, this concept has only had a limited amount of application to humans. This may be because magnetic resonance or CT scans for humans typically provide satisfactory detection of big tumours or other diseases. Because of the small scanning size, optical imaging cannot be the most effective method for detecting metastatic spread in humans. Many clinical-stage businesses are creating NIR imaging methods, mostly for image-guided surgeries [198–200].

### Magnetic resonance imaging

The underlying ideology of MRI seems to be the behaviour of hydrogen nuclei in a magnetic field. Radiofrequency (RF) pulses and magnetic field gradients capture and transform the relaxation of nuclei back to their original oriented states. MRI can examine soft tissues of the body and show full body depth with better resolution without the use of contrasting agents. Furthermore, MRI contrasting agents can further improve MRI signals in two directions: by decreasing the T1-relaxation times of water protons and the T2-relaxation times of water protons.

As a result, there are two main categories of MR contrasting agents: T1 contrasting agents, which are usually paramagnetic complexes (such as complexes of Gd^3+^), and T2 contrasting agents, which are usually NPs made of iron oxide. The enormous surface area of these inorganic NPs enhances contrasting effects. Several MR-contrasting compounds are presently undergoing clinical studies, and several of them have already received approval for use in clinical healthcare situations. MR can offer superior spatial resolution without depth constraints as compared to in vivo fluorescence imaging. MRI has limitations, such as high administration costs and low contrast agent sensitivity. A significant amount of contrasting agent must be supplied in order to attain improved imaging quality. It is difficult to provide such a high amount without toxicity issues [201, 202].

### PET

PET contrasting agents are substances that include radionuclides that release positrons. These substances degrade by emitting positrons, which are subatomic particles with a positive charge. That positron will move just a small distance through the tissues before being annihilated by an electron. Annihilation will then release two rays traveling in opposite directions. Those rays will be captured by detectors nearby and then included in computed images. Generally, radioisotopes such as C-11, N-13, GD-68, Cu-64, Br-76, I-124, and F-18 serve as contrasting agents. The distance travelled by positrons varies depending upon the contrasting agent, and higher-energy positrons often travel a longer distance prior to annihilation, resulting in a greater loss in spatial resolution. The chemical characteristics of radioactive PET contrasting agents are identical during and after radioactive decay, ensuring the stability of the agent’s physiological characteristics that are independent of radioactivity. Since PET is a quantitative method, it is simple to determine how much of a contrasting agent's signal is present in various detected volumes. Nowadays, a combination of MRI and PET imaging equipment has been designed to get more effective result. The PET/MRI combo is among the most therapeutically applicable dual functional imaging probes, resulting in a surge of interest [203–205].

### Ultrasound

A frequently utilized clinical imaging technique for diagnostic purposes is ultrasound (US). The price is inexpensive, operation is uncomplicated, real-time monitoring is possible, and there aren't any safety issues when compared to certain other systems. An ultrasound image is created by the reflection of high-frequency sound waves from the body's internal organs and objects from the transducer against the skin. In areas being observed, US contrast agents concentrate on offering greater resonance frequencies. Almost 20 contrasting compounds have been utilized in clinical trials in this system. Despite the use of these US contrasting agents, resolution remains quite low because resolution and depth are fundamentally opposed to one another [27, 206].

### Photoacoustic tomography (PAT)

By using light-induced pressure waves PAT creates images. A short-pulse laser beam is used to illuminate a target region, and some of the light is absorbed by exogenous or endogenous contrasting agents, converted into heat, and then transformed into pressure using thermoelastic expansion. This pressure increase is perceived by conventional ultrasonic transducers as an audio wave. PAT can simply create multiscale, high-resolution images, decrease optical tissue scattering, have no excitation photons escape into detectors, capture images without speckles, and have a higher signal-to-noise ratio because of a low background. There are instances of PAT in which contrast-enhanced imaging passes through the breast tissue of a chicken at a depth of over 10 cm and at a depth of 5 cm via the human arm. PAT is a newly developed diagnostic method that is giving successful test results in a variety of investigations performed preclinically. Additional clinical studies are currently being conducted [136, 207, 208].

## Challenges in future for biomaterials and theranostics

The main ideas behind how modern nanomaterials used in theranostics are made are the contrast agent, the therapeutic moiety, and the targeting agent. These components were thoroughly researched in order to produce specialized theranostic applications. This discipline has a variety of issues, the main one being the slower translation of test results into practical application. The duration of clinical and preclinical investigations, as well as their massive price and manufacturing problems, frequently influence challenges in this field. Therefore, to advance this area, collaboration and innovation between researchers, engineers, and physicians are essential [137]. More research is needed to optimize physiological barrier permeation while preventing RES, excretion, improving continuous assessment, increasing blood flow time, minimizing the unfavourable side effects of the nanomaterials used, and developing resistance to theranostics. Therefore, ongoing research and advancement are required to use innovative theranostics techniques to address unfulfilled therapeutic requirements [27, 80].

For therapeutic NPs to be used in therapeutic settings, several pressing concerns must first be addressed. Given that each diagnostic and treatment option have its own advantages and disadvantages, it is crucial to pick a combination that maximises the benefits of each. In addition, it may be difficult to tune dose levels and frequency for both therapeutic and imaging agents using a single delivery regimen. Additionally, care must be taken while constructing a nanotheranostic formulation to ensure that neither component (i.e., imaging, and therapeutic moieties) is prematurely released from the delivery system, since this would result in irrelevant or even inaccurate data regarding therapeutic outcomes. Next-generation intelligent delivery systems should be deterministic instead of predictive, with the ability to tune pharmacokinetics and biodistribution so that carriers localize at and only at the intended disease targets and release their therapeutic payload in response to the diseased environment. In addition to therapy and non-invasive monitoring, they should actively look for sites where disease is present, find them, and stay there.

## Conclusion

The most recent advances in the field of theranostic medicine and their potential medicinal applications were covered in this chapter. Theranostic provide an attractive platform due to their ability to support several modalities, such as medications, diagnostics, and stabilizing agents, and to give a whole solution all in one convenient package. Many recent theranostic investigations have focused on orthopaedics, viruses, cardiovascular diseases, and cancer. Thanks to developments in nanotechnology, a plethora of new biomaterials have appeared in recent years. Nowadays, biomaterials are used in theranostic fields as well due to their ability to give local and sustained effect. Theranostics have the potential to become a useful addition to current methods of illness diagnosis and treatment.

## Data Availability

Not applicable.
